# Serum erythropoietin level is increased during stimulation for IVF but not in OHSS

**DOI:** 10.1186/s12958-023-01178-3

**Published:** 2024-01-20

**Authors:** Merituuli Rekola, Kati Korhonen, Leila Unkila-Kallio, Henrik Alfthan, Vedran Stefanovic, Aila Tiitinen, Tomi S. Mikkola, Hanna Savolainen-Peltonen

**Affiliations:** 1 Department of Obstetrics and Gynecology, Helsinki University Hospital and University of Helsinki, PO Box 140, Haartmaninkatu 2, Helsinki, 00290 Finland; 2 HUSLAB, Helsinki University Hospital, Topeliuksenkatu 32, Helsinki, 00029 Finland

**Keywords:** Erythropoietin, In vitro fertilization, Ovarian hyperstimulation syndrome, Angiogenesis, Early pregnancy

## Abstract

**Background:**

Erythropoietin (Epo) is a potent vascular growth factor that induces angiogenesis and antiapoptotic signalling. We investigated whether the development of numerous follicles and corpora lutea during in vitro fertilization (IVF) cycle affects circulating Epo levels and further, if Epo could be used as a novel marker for ovarian hyperstimulation syndrome (OHSS).

**Methods:**

24 women were included in the uncomplicated IVF group and 35 women in the OHSS group. Repeated blood samples from both groups were analysed for Epo, progesterone, blood haemoglobin, and creatinine. Follicular fluid from the IVF group was analysed for Epo and progesterone. Repeated measure analysis was performed for the variables and circulating Epo levels were compared between the IVF group and early OHSS. Furthermore, related growth factors, vascular endothelial growth factor (VEGF) and hypoxia-inducible factor-1 (HIF-1) were analysed from subgroup of women to test for correlation with Epo.

**Results:**

During IVF, circulating Epo increased from natural mid-luteal phase to stimulated mid-luteal phase (median 9.5; 95% CI 7.2–13.4 IU/L and 12.5; 10.3–13.4 IU/L; *p* = 0.003). In cycles resulting in pregnancy, Epo level decreased 14 days after oocyte pick-up (OPU) and remained low thereafter. In cycles not resulting in pregnancy, Epo level increased again 35 days after OPU. Follicle fluid Epo concentration was 1.5 times higher than the serum concentration (median 15.4; 95% CI 10.4–19.2 IU/L vs. 10.2; 8.8–12.7; *p* = 0.006). There was no difference in circulating Epo concentration between early OHSS and uncomplicated IVF. Circulating Epo did not correlate with VEGF or HIF-1.

**Conclusions:**

Circulating Epo levels fluctuate during IVF cycle. We hypothesise this may suggest Epo’s involvement in ovarian physiology and angiogenesis. However, Epo was not a clinical marker for OHSS.

## Background

Erythropoietin (Epo) is a hormone promoting the generation of new red blood cells in the bone marrow. It is mainly produced by the kidney cortices in response to tissue hypoxia [1]. More recently, the recognition of extrarenal sites of Epo production, e.g. the central nervous system and female reproductive organs, has raised interest in the non-hematopoietic effects of Epo, such as angiogenesis and induction of antiapoptotic signalling [2, 3].

Previous studies suggest that circulating Epo levels do not change during the menstrual cycle [4, 5]. However, both endometrial Epo and Epo receptor (Epo-R) mRNA and protein expression fluctuate in different phases of the cycle [6]. Moreover, Epo-induced angiogenesis is regulated by oestrogen in the rodent uterus [7]. Epo mRNA expression has been detected in the ovaries as well, but no data on cyclic changes exist [8]. Finally, Epo and Epo-R mRNA are found more abundantly in malignant tumours of the endometrium and ovaries [9] and Epo concentration in ascites samples of patients with ovarian tumours correlates to the degree of tumour malignancy [10].

Ovarian hyperstimulation syndrome (OHSS) is one of the most serious complications of assisted reproductive technologies. Although the prevention of this iatrogenic complication has taken leaps forward, OHSS is still a continuing concern among clinicians with reported incidence of 2–3% [11]. The syndrome is characterised by massive ovarian enlargement, increased vascular permeability and fluid shift from intravascular to third space compartments inducing abdominal discomfort, nausea, weight gain and dyspnoea. Clinical manifestations include ascites, pleural effusions, haemoconcentration, hypoperfusion of vital organs and oliguria. The diagnosis and classification of OHSS is based on clinical examination, abdominal and transvaginal ultrasound and laboratory tests. Acute kidney injury and thrombosis are severe complications of OHSS. Early OHSS occurs typically within the first week from oocyte pick-up (OPU) and mostly with patients triggered with exogenous human chorionic gonadotropin (hCG). Late OHSS is related to hCG in early pregnancy and occurs 10 or more days after oocyte pick-up [12].

The growth factor best known to associate with OHSS is vascular endothelial growth factor (VEGF), a promoter of angiogenesis and vascular permeability [13]. As Epo has in other contexts proven to be a potent vascular growth factor and a key player in regard to kidney function and pathology, we wanted to investigate its association with IVF and OHSS. Circulating Epo levels during both uncomplicated IVF cycle and OHSS were determined, along with blood haemoglobin levels and kidney function tests. Specifically, the possibility of higher Epo concentrations associating with the development of OHSS was considered.

## Methods

This was a prospective observational study of two groups (IVF and OHSS), performed with informed consent from all the participants and approval from the Helsinki University ethics committee. The data was collected between 2006 and 2008. The original study design and materials have been previously described [14, 15]. In short, for the IVF group, a total of 30 women were originally recruited from Helsinki University hospital’s infertility clinic before the start of IVF treatment. Inclusion criteria were a previously recognized risk factor for OHSS (history of OHSS, polycystic ovary syndrome, BMI < 20 kg/m2 or age < 25) and two existing ovaries without any major surgery. Three patients developed hyperstimulation and were moved to the OHSS group. For the OHSS group, 65 patients presenting with OHSS symptoms were recruited from the hospital’s emergency department. Thirteen cases were excluded (mild OHSS and other IVF complications, primarily infection or bleeding). All the patients underwent repeated blood sampling. In addition, in the IVF group, follicle fluid from OPU was stored if possible (n = 15). General health information from both groups was gathered from medical records and structured questionnaires.

For the present study, cases with available serum samples from at least two separate time points, or both serum and follicle fluid samples from OPU were included. Finally, the IVF group consisted of 24 women and the OHSS group of 35 women (37 cases; 25 early OHSS, 12 late OHSS). Cases were classified as moderate (early n = 19, late n = 2) or severe to critical (early n = 6, late n = 10) using accepted criteria [16]. The patients’ demographic background was similar between the two groups (Table 1). Treatment protocol in the IVF group was the standard at that time: long agonist protocol. In the OHSS group, patients had been treated at private clinics as well and both agonist and antagonist protocols were represented. Both protocols had hCG triggering.

**Table 1:** Background and treatment characteristics

**Background information**		**IVF Group (N = 24)**	**OHSS Group (N = 35)**		***p***
Age (yrs)^a^		34.0 ± 2.6	33.1 ± 3.5		0.26
BMI (kg/m²)^a^		23.6 ± 3.4	22.5 ± 3.2		0.23
Smoking^b^					0.35
	Yes	2 (8.3)	1 (2.9)		
	No	22 (91.7)	34 (97.1)		
Previous OHSS^b^					0.12
	Yes	6 (26.1)	3 (10)		
	No	17 (73.9)	27 (90)		
PCO^b^					0.16
	Yes	6 (26.1)	13 (44.8)		
	No	17 (73.9)	16 (55.2)		
Duration of infertility (yrs)^a^		4.9 ± 2.9	4.1 ± 2.3		0.28
AMH (µg/L)^*c,d*^		3.6 (3.0-4.1)	2.7 (1.9–4.2)		0.25
**Treatment characteristics**		**IVF Group (N = 24)**	**Early (N = 25)**	**Late (N = 12)**	
Protocol^b^					< 0.05
	Agonist	24 (100)	17 (68)	9 (75)	
	Antagonist	0 (0)	8 (32)	3 (25)	
FSH starting dose (IU)^*c*^		150 (150–200)	150 (150–225)	150 (150–250)	0.69
FSH cumulative dose (IU)^*c*^		1650 (1650–2000)	1375 (1200–1600)	1688 (1500–2400)	0.08
Number of follicles^c^		17 (14–26)	38 (29–48)	21 (21–25)	< 0.001
E2 OPU2 (nmol/L)^*c*^		3.7 (2.1–4.2)	NA	NA	NA
E2 Admission (nmol/L)^*c,e*^		NA	9.7 (7.2–13.5)	7.4 (6.5–10.1)	0.44
E2 OPU7 (nmol/L)^*c*^		3.0 (2.3–4.1)	8.6 (5.9–11.8)	NA	< 0.001
Luteal support^b^					0.19
	Vaginal progesterone	24 (100)	15 (100)	11 (91.7)	
	hCG	0 (0)	0 (0)	1 (8.3)	
Embryo transfer^b^					< 0.001
	None	0 (0)	10 (40)	0 (0)	
	One embryo	23 (95.8)	14 (56)	9 (75)	
	Two embryos	1 (4.2)	1 (4)	3 (25)	
Pregnancy^b^					< 0.001
	Yes	12 (50)	6 (24)	12 (100)	
	No	12 (50)	19 (76)	0 (0)	

BMI = body mass index, PCO = polycystic ovaries, AMH = anti-Müllerian hormone, FSH = follicle stimulating hormone, E2 = oestradiol, OPUn = days from ovarian pick-up, hCG = human chorionic gonadotropin. NA = not applicable. The data are expressed as ^a^mean ± standard deviation for parametric variables, ^b^number of women (percentage) for categorical variables or ^c^median (95% confidence interval) for non-parametric variables. ^d^AMH was analysed at OPU7. ^e^Median day for admission was OPU4 for early OHSS and OPU14 for late OHSS. Group differences were analysed with independent samples T-test test for parametric variables, Kruskal-Wallis or Mann-Whitney U test for non-parametric variables and chi-square test for categorical parameters

In the IVF group, blood samples were collected during clinic visits and arranged according to oocyte pick-up date (OPUn). Samples were taken at mid-luteal phase before the start of agonist therapy, at ovarian pick-up (OPU), stimulated mid-luteal phase (OPU7), time of pregnancy test (OPU14) and check-up (OPU35).

Blood samples from the OHSS patients were collected on admission to the emergency clinic and repeatedly on the ward, at discharge, and during a voluntary follow-up a week from discharge. Individual worst day of symptoms was determined by clinical criteria (presentation of dyspnoea, fever, oliguria, maximum waist circumference, weight, and drainage of ascites or pleural fluid). For early OHSS, median day of admission was OPU4 and median worst day of symptoms OPU6. For late OHSS, respective days were OPU14 and OPU16.

Blood haemoglobin (B-Hb), serum creatinine (reference value 50–90 µmol/l), serum oestradiol (E2), and human choriogonadotropin (hCG) were analysed routinely for clinical use by accredited methods (HUSLAB, Helsinki University Hospital laboratory) in both groups. Samples were centrifuged and stored at -80˚C for later analysis.

Serum erythropoietin was quantitated with immunochemiluminometric assay on the IMMULITE 2000XPi analyser (Siemens Healthcare Diagnostics, Llanberis, United Kingdom). The detection limit for the assay was 1.0 IU/L. Within-run coefficient of variation (CV) was < 7% in the concentration range of 4–615 IU/L. Total CV was < 10% in the same concentration range. The reference value for serum erythropoietin in adults is 5.4–21.8 IU/L.

Progesterone was quantitated with an electrochemiluminometric assay on a cobas e411 analyzer (Progesterone III, Roche Diagnostics, Mannheim, Germany). Detection limit was 0.2 nmol/L. Inter-assay coefficient of variation was 11.8% at 0.7 nmol/L, 3.3% at 2.3 nmol/L, 2.5% at 9.5 nmol/L and 1.2% at 164 nmol/L, respectively.

Anti-Müllerian hormone (AMH) was analysed by an ultrasensitive AMH ELISA (AnshLabs®, Webster, TX, USA) in collaboration with the Helsinki University Hospital Laboratory (HUSLAB). Detection limit was 0.023 µg/L and the intra- and inter-assay CV were ≤ 4.0% and < 4.8%, respectively [15].

As a previously recognized growth factor associated with OHSS, VEGF was analysed from a subgroup of 15 women with remaining samples (five from the IVF group and 10 from the OHSS group) to look for possible correlation with Epo. VEGF was quantitated with enzyme-linked immunoassay (Human VEGF Assay Kit, IBL International GmbH, Hamburg, Germany). Measurement range was 15.63–1000 pg/mL and inter-assay CV was 8.5% at 22.60 pg/mL, 9.4% at 84.31 pg/mL and 7.0% at 338.25 pg/mL, respectively. Finally, as hypoxia is known to stimulate Epo synthesis, hypoxia-inducible factor-1 (HIF-1), was analysed from plasma samples of 15 women in the IVF group and 10 women in the OHSS group by enzyme-linked immunoassay (Invitrogen™ HIF-1 Alpha ELISA Kit, Bender MedSystems GmbH, Vienna, Austria). Detection limit was 30 pg/mL and the intra- and inter-assay CV < 10% each in the concentration range of 81.92–20,000 pg/L.

### Statistics

Data analyses were run with IBM® SPSS® Statistics version 26. Shapiro-Wilk’s test was used for the evaluation of value distribution. Differences between groups were analysed with independent samples T-test for parametric variables, Mann-Whitney U and Kruskal-Wallis test for non-parametric variables and Chi-square test for categorical variables. Friedman’s test and Wilcoxon signed-rank test were used for non-parametric repeated measure analyses. For parametric variables, repeated measure ANOVA (Bonferroni, Greenhouse-Gessler correction) was used. Correlations were analysed with Spearman’s test. Results are presented in median with 95% confidence interval (CI) for non-parametric variables and mean ± standard deviation (SD) for parametric tests. The level of significance was *p* < 0.05.

## Results

### Circulating and follicular fluid Epo during the IVF cycle

**Fig. 1:**
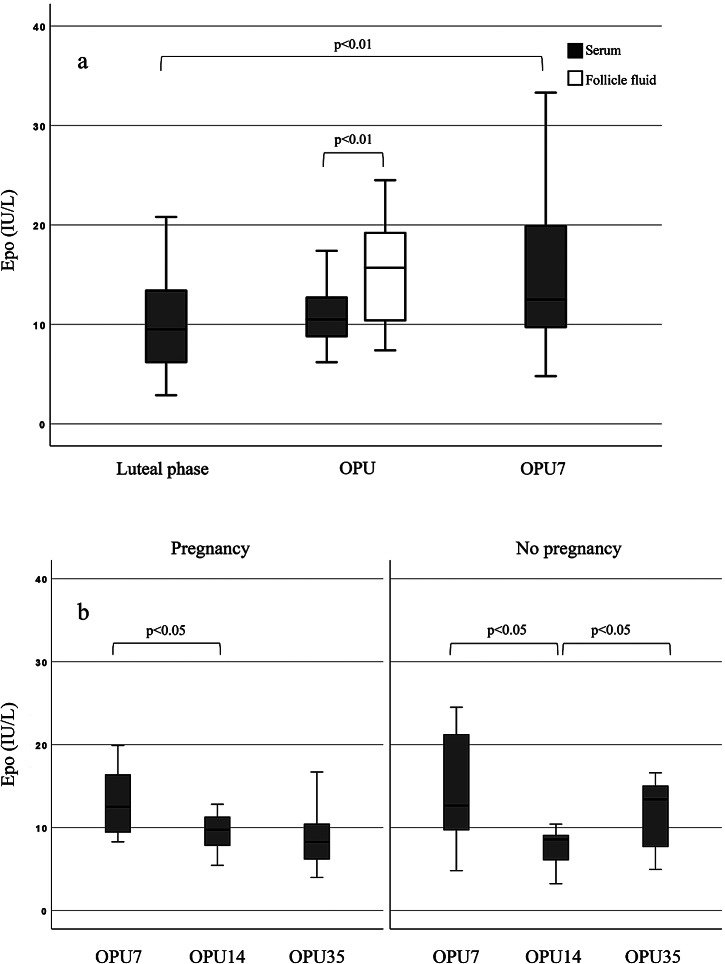
Serum erythropoietin (Epo) in uncomplicated in vitro fertilization cycles from **(a)** natural mid-luteal phase to stimulated luteal phase seven days from ovarian pick-up (OPU) and follicular fluid Epo at OPU, and **(b)** serum erythropoietin seven to 35 days from OPU by pregnancy status. OPUn = days from OPU

In the IVF group, circulating Epo level was higher at OPU7 (12.5; 95% CI 10.3–13.4 IU/L) than at natural cycle mid-luteal phase (9.5; 7.2–13.4 IU/L; *p* = 0.003; pairwise analysis) (Fig. 1a). Likewise, serum progesterone was higher at OPU7 (122.5; 84.1–228.0 nmol/L) than in the natural cycle (18.1; 1.5–36.9 nmol/L; *p* = 0.005). Serum creatinine level remained stable during the cycle (*p* = 0.17). Mean B-Hb decreased from natural (133±9 g/L) to stimulated mid-luteal phase (126±10 g/L, *p* = 0.003) but was stable thereafter.

From OPU7, cycles resulting in pregnancy or not were analysed separately. In the pregnant group, serum Epo level decreased from OPU7 to OPU14 (12.5 vs. 9.7 IU/L; *p* = 0.036) and remained low during follow-up. By contrast, in cycles not resulting in pregnancy, serum Epo level decreased from OPU7 to OPU14 (12.7 vs. 8.6; *p* = 0.012) but increased again at OPU35 (13.4 IU/L; *p* = 0.028) (Fig. 1b). The change from OPU14 to OPU35 was significantly different between the two groups (ΔEpo − 2.1; 95% CI -4.4–1.8 IU/L vs. 3.3; 1.3–7.7 IU/L; *p* = 0.013). Serum progesterone was steady in the pregnant group from OPU7 (104.4 nmol/L) to OPU35 (173.8 nmol/L; *p* = 0.31), whereas levels rapidly decreased from OPU7 to OPU35 (147.3 vs. 12.6 nmol/L; *p* = 0.004) in the cycles not resulting in pregnancy. There was no correlation between serum hCG and Epo at OPU14. Serum progesterone correlated positively with circulating Epo at OPU7 (r = 0.786, *p* = 0.021) in cycles resulting in pregnancy.

Follicle fluid Epo level was 1.5 times higher than in the corresponding serum sample (15.4; 95% CI 10.4–19.2 IU/L vs. 10.2; 8.8–12.7; *p* = 0.006) (Fig. 1a). Follicle fluid and serum Epo levels did not correlate with each other (r = 0.42, *p* = 0.13). Neither serum Epo at OPU nor follicle fluid Epo correlated with the number of follicles (r = 0.167, *p* = 0.55). Follicle fluid progesterone level was notably, 290 times, higher than in the serum (63 695; 95% CI 53 950–81 220 nmol/L vs. 33.7; 28.3–55.6 nmol/L; *p* = 0.001). Follicle fluid Epo and progesterone had no correlation (*p* = 0.96).

Serum Epo did not correlate with serum vascular endothelial growth factor (VEGF) (data not shown) or HIF-1 at natural mid-luteal phase (r=-0.320, *p* = 0.29), OPU (r = 0.265, *p* = 0.53), or OPU7 (r=-0.504, *p* = 0.079). HIF-1 did not change from natural to stimulated mid-luteal phase (data not shown). Furthermore, there was no correlation between follicular fluid Epo and HIF-1 (r=-0.682, *p* = 0.062).

### Circulating Epo in OHSS

**Fig. 2:**
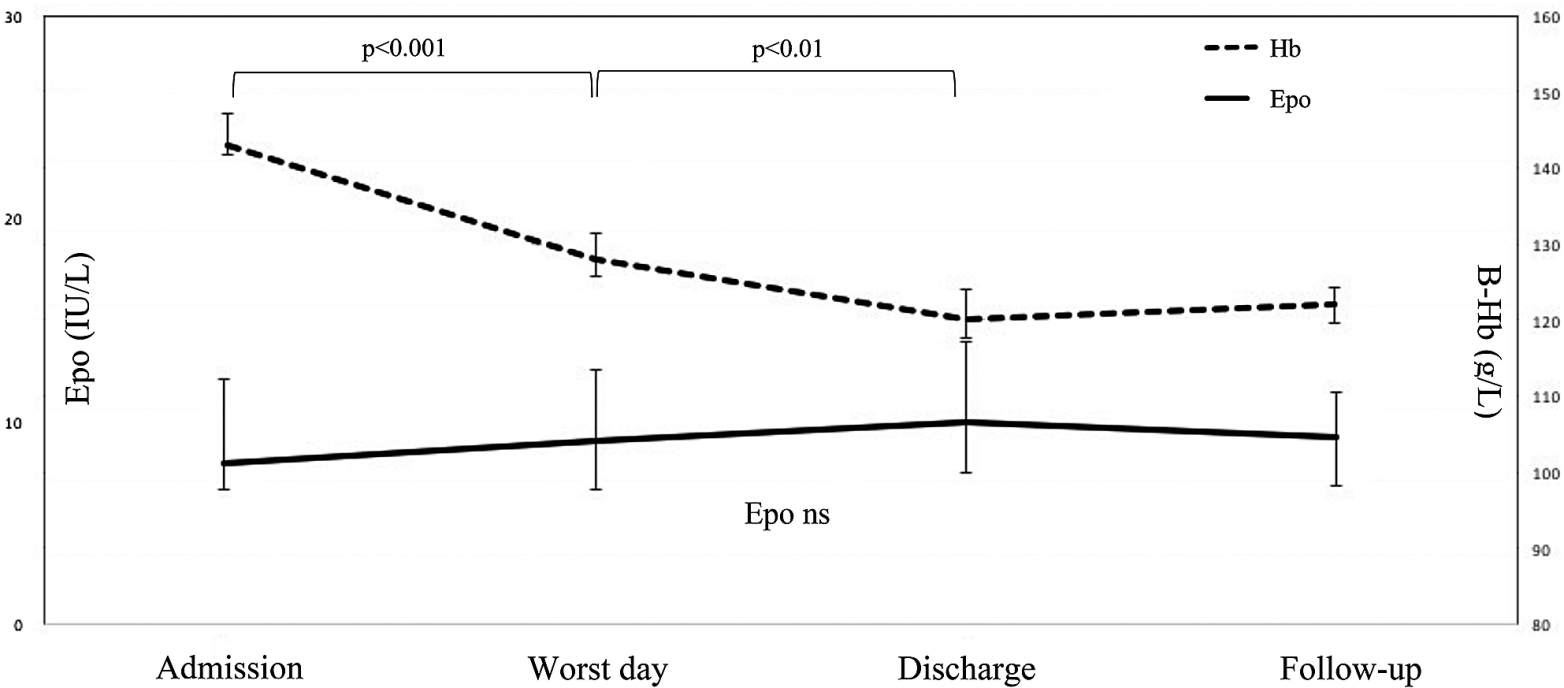
Median serum erythropoietin (Epo) (95% confidence interval) and median blood haemoglobin (B-Hb) level (95% CI) in ovarian hyperstimulation syndrome from admission to the emergency department until follow-up. Ns = non-significant

There were no changes in serum Epo between admission, symptomatically worst day, discharge and follow-up in the OHSS group (*p* = 0.19) (Fig. 2). Circulating Epo and progesterone levels correlated negatively on admission (r=-0.50, *p* = 0.022) and at follow-up (r=-0.38, *p* = 0.037).

In early OHSS, serum Epo level was lower on admission than at discharge (8.8 vs. 13.0 IU/L; *p* = 0.032). In late OHSS, circulating Epo level was stable but significantly lower than in early OHSS at discharge and follow-up. (Table 2) Pregnancy did not affect Epo levels in early OHSS. Furthermore, there was no difference (*p* = 0.74) in circulating Epo between the IVF group at OPU7 (12.5; 95% CI 10.3–13.4 IU/L) and early OHSS group on the worst day of symptoms (median OPU6; 12.0; 8.8–17.2 IU/L).

**Table 2:** Serum erythropoietin and related laboratory measures in patients with ovarian hyperstimulation syndrome

	Early n = 25 Late n = 12	Admission n = 27	Worst day n = 28	Discharge n = 32	Follow-up n = 32	*p*
S-Epo (IU/L)^a^	Early n = 13	8.8 (6.8–14.0)	12.0 (8.8–17.2)	13.0 (8.8–19.4)*	10.4 (9.1–12.6)**	0.53
	Late n = 5	5.2 (3.8–8.5)	7.2 (5.1-9.0)	7.2 (3.6–10.0)*	6.5 (4.8–8.9)**	0.13
S-Prog (nmol/L)^a^	Early n = 12	728.2 (646.1–1071.0)*	534.6 (411.0-923.7)*	153.0 (52.5–442.0)***	37.2 (1.8–48.6)***	< 0.001
	Late n = 3	1399.5 (707.1–1877.0)*	909.0 (885.2–1557.0)*	811.2 (753.4–1083.0)***	1022.5 (841.3–1142.0)***	N.D
B-Hb (g/L)^a^	Early n = 21	143 (133–150)	127 (121–133)	118 (113–124)	123 (119–129)	< 0.001
	Late n = 10	143 (122–165)	134 (113–145)	121 (118–137)	112 (108–131)	< 0.001
SCr (µmol/L)^b^	Early n = 17	64±12	60±10	56±6	57±10	< 0.01
	Late n = 9	65±13	56±9	55.5±7	54±7	< 0.01

S = serum, Epo = erythropoietin, Prog = progesterone, B-Hb = blood haemoglobin, SCr = serum creatinine, N.D = Not done (n < 5). The data are expressed as ^a^ median (95% confidence interval) for non-parametric variables and ^b^ mean ± standard deviation for parametric variables. Repeated measure analyses (minimum five samples available) were conducted with Friedman’s test for non-parametric variables and repeated measure ANOVA for parametric tests. P-value is for trend. Group differences between early and late OHSS were analysed with independent samples T-test test for parametric variables and Mann-Whitney U test for non-parametric variables. Differences between the groups are presented in the index: *p* *≤0.05, *p* **≤0.01, *p* ***≤0.001

In both early and late OHSS, serum creatinine decreased from admission to discharge (*p* = 0.001 and *p* = 0.003 respectively), although the mean values remained within reference limits (Table 2). Serum creatinine and Epo correlated negatively at discharge (r=-0.36, *p* = 0.044) but not at other time points. Overall, four cases out of the 37 (10.8%) presented with markedly affected renal function by either elevation of serum creatinine or oliguria. Their circulating Epo levels did not differ from the rest of the OHSS cases at admission, worst day, discharge or follow-up (*p* = 0.20, 0.58, 0.53 and 0.12, respectively).

B-Hb level decreased from admission to discharge in both early and late OHSS (*p* < 0.001 each) (Table 2), consistent with fluid administration on the ward. Serum Epo correlated negatively with B-Hb concentration on admission (r=-0.40, *p* = 0.038) and on the worst day of symptoms (r=-0.43, *p* = 0.023) (Fig. 2). Serum Epo did not correlate with VEGF (r=-0.200, *p* = 0.70 for worst day of symptoms and r = 0.636, *p* = 0.66 for follow-up) or HIF-1 (r=-0.866, *p* = 0.33 for worst day of symptoms and r=-0.051, *p* = 0.94 for follow-up).

## Discussion

In contrast to studies on regular menstrual cycles, where circulating Epo levels have been stable [4, 5], we demonstrate an increase in serum Epo from natural to stimulated mid-luteal phase. Similar changes have been reported for VEGF, a well-recognized growth factor involved in both normal ovarian physiology and the development of OHSS [13]. To our knowledge, this was the first time circulating or follicular fluid Epo was studied in IVF or OHSS.

Follicular fluid Epo concentration was significantly higher than in the serum and did not correlate with the corresponding serum sample, supporting the idea of a paracrine Epo/Epo-R system contributing to the development and maturation of follicles and related angiogenesis in the ovaries. In fact, immunohistochemical analyses have shown Epo-R protein to be present not only in the endothelium of blood vessels and endothelial cells of the endometrium but in follicles of different stages, including luteal cells [8]. Furthermore, although Epo production in the kidneys is induced by tissue hypoxia and primarily mediated by HIF-1 [3], in the uterus Epo production appears to be upregulated rather by oestrogen than tissue hypoxia [7]. In our study, HIF-1 did not explain changes in circulating or follicular fluid Epo further building to the hypothesis of a similar hormone-mediated system at play in the maturation of follicles and function of corpus luteum.

VEGF and fibroblast growth factor are mediators known to contribute to luteal angiogenesis. VEGF immunoneutralization has markedly reduced endothelial cell proliferation in primates [17], and the neutralization of either resulted in decreased progesterone production in the cow [18]. The increase in circulating VEGF documented for IVF cycles around OPU is clearly proceeded by the administration of hCG [19]. In our study, there was no correlation between hCG and Epo. A positive correlation between serum Epo and progesterone level was detected in the pregnant group at OPU7 but not in the non-pregnant group, nor was any correlation found at OPU14 or OPU35 in either subgroup. Hence changes in circulating progesterone do not seem to predict subjacent fluctuating in Epo levels or vice versa.

A notable finding was the contribution of pregnancy. Circulating Epo decreased from OPU7 to OPU35 in cycles resulting in pregnancy in contrast to cycles that did not. One possible explanation is that at OPU35, most of the women in the non-pregnant group had already reached their next natural luteal phase. Epo response to anaemia has been found to be dampened in the first two trimesters of pregnancy [20], but its behaviour in such early pregnancy has not been described in any studies that could be found.

Haemoglobin level slightly decreased from natural mid-luteal phase to stimulated mid-luteal phase but remained stable thereafter. Despite more substantial changes in B-Hb in the OHSS group due to intravenous fluid administration, Epo was practically steady. Furthermore, as both serum Epo concentration and B-Hb level were within their physiological range, their connection did not seem to account for our results. Serum creatinine remained unchanged during the IVF cycle and does not offer any explanation to the changes in circulating Epo either.

Our hypothesis of higher Epo levels associating with the development of OHSS was not supported: there was no difference in Epo levels between early OHSS cases and the IVF group at OPU7, and further, Epo levels were stable during recovery in the OHSS group.

The strengths of this study include its novelty in demonstrating changes in circulating Epo during uncomplicated IVF as well as in OHSS. Different phases of the IVF cycle were covered with repeated blood samples. Furthermore, a good variety of possible contributing factors (kidney function, changes in haemoglobin, progesterone and hCG levels) were considered. However, the reported changes in circulating Epo are from a clinical perspective small, compared to the potential 1000-time elevation in circulating Epo seen in cases of extreme anaemia or haemorrhage. Further limitations are missing data due to insufficient serum samples and missed clinic visits. That said, participation rate for the voluntary follow-up visit was good: 18/24 (75%) for the IVF group and 33/37 (89%) for the OHSS group. Behaviour of circulating Epo before the onset of OHSS symptoms was not investigated as these patients were only recruited from the emergency department.

## Conclusions

In conclusion, our study indicates that in contrast to the regular menstrual cycle, circulating Epo changes during stimulation for IVF. The results support Epo’s involvement in ovarian angiogenesis during the luteal phase.

## Data Availability

The datasets analysed during the current study are available from the corresponding author on reasonable request.

## Abbreviations

AMH: Anti-Müllerian hormone
B-Hb: Blood haemoglobin
E2: Oestradiol
Epo: Erythropoietin
Epo-R: Erythropoietin receptor
hCG: Human choriogonadotropin
HIF-1: Hypoxia-inducible factor-1
IVF: In vitro fertilization
OHSS: Ovarian hyperstimulation syndrome
OPU: Ovarian pick-up
VEGF: Vascular endothelial growth factor
